# Desvendando as Controvérsias da Dissecção Aórtica Tipo B – Interpretando as Evidências

**DOI:** 10.36660/abc.20230550

**Published:** 2023-08-28

**Authors:** Walter J. Gomes, Eduardo N. Gomes, Nelson A. Hossne

**Affiliations:** 1 Universidade Federal de São Paulo Escola Paulista de Medicina Disciplina de Cirurgia Cardiovascular São Paulo SP Brasil Escola Paulista de Medicina da Universidade Federal de São Paulo (EPM/UNIFESP) – Disciplina de Cirurgia Cardiovascular, São Paulo, SP – Brasil; 2 Hospital Regional de Sorocaba “Dr. Adib Domingos Jatene” Sorocaba SP Brasil Hospital Regional de Sorocaba “Dr. Adib Domingos Jatene”, Sorocaba, SP – Brasil

**Keywords:** Doenças da Aorta/complicações, Aneurisma Dissecante/cirurgia, Procedimentos Endovasculares, Remodelação Aórtica

Diferentemente da dissecção aórtica tipo A, múltiplos aspectos da história natural da disseção aórtica tipo B (DATB) permanecem obscuros e indefinidos, resultando em incertezas em seu prognóstico, recomendações e manejo.^1,2^ Entre os fatores críticos que impactam os desfechos da DATB, a trombose do falso lúmen parece ser um achado associado que exibe uma correlação sólida com o prognóstico tardio e trazendo implicações clínicas significativas. Além de ser um indicador prognóstico crucial para pacientes com DATB, a presença ou ausência de trombose do falso lúmen tem sido empregada para guiar as decisões de tratamento, afetando a escolha de intervenções terapêuticas, como o reparo aórtico endovascular torácico (TEVAR) e ajustando o seguimento pós-tratamento.^3^

Nesse sentido, Tang et al.,^4^ relatam neste fascículo um aspecto interessante da correlação entre imagens e clínica. Analisando uma grande coorte de pacientes consecutivos com DATB, eles investigaram os fatores que afetam a trombose do falso lúmen aórtico em pacientes com DATB, focando em compreender o papel da morfologia aórtica na trombose do falso lúmen. Imagens de angiografia tomográfica computadorizada foram utilizadas para as medidas. Um software especializado reconstruiu modelos tridimensionais da disseção aórtica, e medidas dos diâmetros do lúmen verdadeiro e falso foram realizadas em diferentes zonas da aorta. A incidência de falso lúmen trombótico foi maior em pacientes mais idosos com função renal normal do que em pacientes mais jovens ou com função renal comprometida.^4^

Além disso, o diâmetro do lúmen verdadeiro da aorta descendente mostrou-se relacionado à trombose no falso lúmen. Especificamente, quando o diâmetro do lúmen verdadeiro era maior que o diâmetro do falso lúmen, as condições favoreciam a trombose no falso lúmen, enquanto o cenário oposto resultava em um falso lúmen com mais perviedade. Compreender esses fatores do processo de trombose pode ajudar na conduta em pacientes com DATB submetidos a tratamento com TEVAR.^5,6^

O estudo concluiu que o diâmetro do lúmen verdadeiro na aorta descendente e a função renal são fatores importantes que influenciam a ocorrência de trombose do falso lúmen em pacientes com DATB. No entanto, o estudo possui algumas limitações, como ser um estudo retrospectivo de único centro com tamanho de amostra limitado e a necessidade de analisar mais características morfológicas em estudos prospectivos mais abrangentes. Os achados também indiretamente apoiam o uso do TEVAR como opção de tratamento para DATB, sugerindo que o TEVAR pode efetivamente induzir a trombose do falso lúmen e melhorar os resultados do paciente. A trombose do falso lúmen tem sido correlacionada com um prognóstico tardio favorável com DATB. O mecanismo provavelmente envolve a estabilização do processo de remodelação aórtica e a redução do risco de complicações, como a ruptura aórtica.^7,8^

Na verdade, a trombose do falso lúmen não elimina completamente o risco de complicações e eventos tardios, e o aumento do falso lúmen ou eventos de reentrada persistem mesmo na presença de trombose. Esses eventos podem levar a disseção recorrente ou desenvolvimento de aneurismas aórticos, ressaltando a necessidade de monitoramento em longo prazo em pacientes com DATB.^3,9^

Entretanto, esse benefício tem sido contestado por evidências adicionais, com metanálises e revisões sistemáticas revelando que a trombose parcial, cujos efeitos protetores foram sugeridos primeiramente por Tsai et al.,^7^ em 2007, não está associado a uma taxa de expansão mais rápida do diâmetro aórtico.^7,10^

Permanece um debate continuado sobre a melhor estratégia de tratamento para a DATB.^11^ As opções incluem o tratamento clínico com controle da pressão arterial e monitoramento regular, o reparo endovascular (TEVAR) ou a cirurgia aberta. Determinar a abordagem mais adequada depende de diversos fatores, como o grau da disseção, a presença de complicações e o estado geral do paciente. O momento da intervenção tem sido outro ponto significativo de controvérsia. A intervenção precoce (TEVAR ou cirurgia aberta) é recomendada para prevenir complicações e reduzir o risco de ruptura ou extensão da dissecção, enquanto outros especialistas defendem uma abordagem mais conservadora com tratamento clinico inicialmente, reservando a intervenção para casos com complicações ou sintomas refratários. Diretrizes respeitáveis recomendam a terapia clínica isolada para DATB não complicada, reservando o TEVAR para casos complicados. Embora o TEVAR tenha mostrado resultados promissores em alguns estudos, preocupações têm sido levantadas sobre complicações potenciais, como endoleaks ou problemas relacionados à endoprótese (stent) em longo prazo. Por outro lado, a cirurgia aberta pode apresentar maior risco inicial, mas pode fornecer resultados mais duradouros.^9,12^

Alguns estudos buscaram determinar se a intervenção endovascular precoce poderia reduzir o risco de complicações futuras ou remodelação aórtica adversa em comparação com o tratamento clínico otimizado (TCO), especialmente em pacientes com características de alto risco. O estudo ADSORB comparou o TCO vs. TEVAR mais TCO; não foram evidenciadas mortalidade precoces em ambos os grupos e, no acompanhamento de 1 ano, apenas um óbito ocorreu, no grupo TEVAR. O TEVAR foi superior ao TCO isolado mostrando diferenças significativas na trombose parcial ou ausente do falso lúmen, dilatação aórtica e ruptura; no entanto, os principais benefícios clínicos permanecem incertos em longo-prazo.^13^ No estudo INSTEAD-XL, em pacientes com disseção aórtica tipo B não complicada, o TEVAR profilático mais TCO foi associado a uma melhora na sobrevida relacionada a eventos aórticos em 5 anos e na progressão tardia da doença. Não houve benefício significativo na mortalidade por todas as causas.^14^

Portanto, o papel do TCO isolado versus intervenção precoce (TEVAR ou cirurgia) em pacientes com DATB não complicada permanece incerto. Pesquisas adicionais são necessárias para identificar subgrupos de pacientes que podem se beneficiar mais da intervenção precoce ou do manejo conservador; a natureza complexa e dinâmica da disseção aórtica requer avaliação individualizada do paciente e monitoramento contínuo tardio.^15^

Como parte da comunidade cardiovascular na linha de frente do tratamento dessa doença potencialmente letal, nós ainda aguardamos evidências mais robustas para apoiar as recomendações que proporcionem aos pacientes a conduta adequada provendo o melhor prognóstico em longo prazo.
