# Supplementary material for: Desvendando as Controvérsias da Dissecção Aórtica Tipo B – Interpretando as Evidências
Source: Arq Bras Cardiol. 2023 Aug 28;120(8):e20230550. [Article in Portuguese] doi: 10.36660/abc.20230550 (PMC10519226; doi:10.36660/abc.20230550)
Supplement: Translation

# Translation: Unfolding Type B Aortic Dissection Controversies – Piecing Together the Evidence

**Authors:** Walter J. Gomes, Eduardo N. Gomes, Nelson A. Hossne

**Affiliations:** 1 São Paulo SP Brazil Escola Paulista de Medicina da Universidade Federal de São Paulo (EPM/UNIFESP) – Disciplina de Cirurgia Cardiovascular, São Paulo, SP – Brazil; 2 Sorocaba SP Brazil Hospital Regional de Sorocaba “Dr. Adib Domingos Jatene”, Sorocaba, SP – Brazil

**Keywords:** Aortic Disease/complications, Aneurysm, Dissecting/surgery, Endovascular Procedures, Aortic Remodeling

Unlike type A aortic dissection, many aspects of the natural history of type B aortic dissection (TBAD) remain blurred and unsettled, leading to uncertainties in its prognosis, recommendations, and management.^1,2^ Among the critical factors impacting TBAD outcomes, the thrombosis of the false lumen seems to be an associated finding exhibiting a robust correlation with late prognosis and conveying significant clinical implications. In addition to being a critical prognostic indicator for TBAD patients, the presence or absence of false lumen thrombosis has been employed to guide treatment decisions, affecting the choice of therapeutic interventions, the thoracic endovascular aortic repair (TEVAR) and adjusting post-treatment follow-up.^3^

In this way, Tang et al.^4^ report an interesting aspect of the correlation between imaging and clinics in this issue. Analyzing a large cohort of consecutive patients with TBAD, they investigated the factors affecting false lumen thrombosis in patients with TBAD, focusing on understanding the role of aortic morphology in the thrombosis of the false lumen. Computed tomographic angiography images were used for measurements. Specialized software reconstructed three-dimensional models of aortic dissection, and measurements of true and false lumen diameters were performed at different zones of the aorta. The incidence of thrombotic false lumen was higher in older patients with normal renal function than in younger patients or those with compromised kidney function.^4^

Additionally, the diameter of the true lumen of the descending aorta was found to be related to the thrombosis in the false lumen. Specifically, when the true lumen diameter was larger than the false lumen diameter, conditions favored thrombosis in the false lumen, while the opposite scenario resulted in a more patent false lumen. Understanding these factors may help in predicting and managing the thrombosis process in TBAD patients undergoing TEVAR treatment.^5,6^

The study concluded that the diameter of the true lumen in the descending aorta and renal function are important factors influencing the occurrence of false lumen thrombosis in patients with TBAD. However, the study acknowledged some limitations, such as being a single-center retrospective study with limited sample size and the need for more morphological features in larger prospective studies. The findings also indirectly supported using TEVAR as a treatment option for TBAD, suggesting that TEVAR can effectively induce false lumen thrombosis and improve patient outcomes. The thrombosis of the false lumen has been correlated with a favorable late prognosis with TBAD. The mechanism likely involves stabilizing the aortic remodeling process and reducing the risk of complications, such as aortic rupture.^7,8^

False lumen thrombosis does not eliminate the risk of complications and late events, and false lumen enlargement or re-entry tear events persist even in thrombosis. These events can lead to recurrent dissection or the development of aortic aneurysms, highlighting the need for long-term surveillance in TBAD patients.^3,9^

Furthermore, this benefit has been challenged by further evidence, with meta-analysis and systematic review revealing that partial thrombosis, whose protective effects were first suggested by Tsai et al.,^7^ is not associated with a faster aortic growth rate.^7,10^

There is an ongoing debate over the best treatment strategy for TBAD.^11^ Options include medical management with blood pressure control and close monitoring, endovascular repair (TEVAR), or open surgical repair. Determining the most appropriate approach depends on various factors, such as the dissection extension, the presence of complications, and the patient's general health. The timing of intervention has represented another significant point of dispute. Early intervention (either TEVAR or open surgery) is advocated to prevent complications and reduce the risk of rupture or extension, while others argue for a more conservative approach with medical management first, reserving intervention for cases with complications or refractory symptoms. Authoritative guidelines recommend medical therapy alone for non-complicated TBAD, reserving TEVAR for complicated cases. While TEVAR has shown promising results in some studies, concerns have been raised about potential complications, such as endoleaks or stent graft-related issues in the long run. Open surgical repair, on the other hand, may have a higher initial risk but could provide more durable results.^9,12^

Several trials aimed to determine if early endovascular intervention might reduce the risk of downstream complications or adverse aortic remodeling compared to optimal medical treatment (OMT), particularly in patients with high-risk features. The ADSORB trial compared OMT vs. TEVAR plus OMT; no early deaths were found in either group, and, at 1-year follow-up, just one fatality occurred in the TEVAR group. With significant differences in partial or no false-lumen thrombosis, aortic dilatation, and rupture, TEVAR was superior to OMT alone; however, the main clinical benefits remain uncertain.^13^ In the INSTEAD-XL trial, prophylactic TEVAR plus OMT was associated with improved 5-year aorta-specific survival and delayed disease progression in patients with uncomplicated type B aortic dissection. No significant benefit was seen in all-cause mortality.^14^

Therefore, the role of OMT alone versus early intervention (TEVAR or surgery) in patients with uncomplicated TBAD remains uncertain. Further research is needed to identify subgroups of patients who may benefit most from early intervention or conservative management; aortic dissection's complex and dynamic nature requires individualized patient assessment and ongoing monitoring.^15^

As part of the cardiovascular community at the forefront of treating this deadly disease, we still await more robust evidence for endorsing recommendations to provide patients with the best possible management and long-term prognosis.
